# Environmental Chemicals in Pregnant Women in the United States: NHANES 2003–2004

**DOI:** 10.1289/ehp.1002727

**Published:** 2011-01-14

**Authors:** Tracey J. Woodruff, Ami R. Zota, Jackie M. Schwartz

**Affiliations:** Program on Reproductive Health and the Environment, Department of Obstetrics, Gynecology and Reproductive Sciences, University of California, San Francisco, Oakland, California, USA

**Keywords:** chemicals, environmental exposures, NHANES, pregnancy

## Abstract

**Background:**

Exposure to chemicals during fetal development can increase the risk of adverse health effects, and while biomonitoring studies suggest pregnant women are exposed to chemicals, little is known about the extent of multiple chemicals exposures among pregnant women in the United States.

**Objective:**

We analyzed biomonitoring data from the National Health and Nutritional Examination Survey (NHANES) to characterize both individual and multiple chemical exposures in U.S. pregnant women.

**Methods:**

We analyzed data for 163 chemical analytes in 12 chemical classes for subsamples of 268 pregnant women from NHANES 2003–2004, a nationally representative sample of the U.S. population. For each chemical analyte, we calculated descriptive statistics. We calculated the number of chemicals detected within the following chemical classes: polybrominated diphenyl ethers (PBDEs), perfluorinated compounds (PFCs), organochlorine pesticides, and phthalates and across multiple chemical classes. We compared chemical analyte concentrations for pregnant and nonpregnant women using least-squares geometric means, adjusting for demographic and physiological covariates.

**Results:**

The percentage of pregnant women with detectable levels of an individual chemical ranged from 0 to 100%. Certain polychlorinated biphenyls, organochlorine pesticides, PFCs, phenols, PBDEs, phthalates, polycyclic aromatic hydrocarbons, and perchlorate were detected in 99–100% of pregnant women. The median number of detected chemicals by chemical class ranged from 4 of 12 PFCs to 9 of 13 phthalates. Across chemical classes, median number ranged from 8 of 17 chemical analytes to 50 of 71 chemical analytes. We found, generally, that levels in pregnant women were similar to or lower than levels in nonpregnant women; adjustment for covariates tended to increase levels in pregnant women compared with nonpregnant women.

**Conclusions:**

Pregnant women in the U.S. are exposed to multiple chemicals. Further efforts are warranted to understand sources of exposure and implications for policy making.

Exposure to chemicals during fetal development can increase the risk of adverse health consequences, including adverse birth outcomes (e.g., preterm birth and birth defects), childhood morbidity (e.g., neurodevelopmental effects and childhood cancer), and adult disease and mortality (e.g., cancer and cardiovascular effects) (Gluckman and Hanson 2004; Stillerman et al. 2008). Biomonitoring studies report nearly ubiquitous exposure to many chemicals in the U.S. population—for example, bisphenol A (BPA), perchlorate, and certain phthalates and polybrominated diphenyl ethers (PBDEs) [Centers for Disease Control and Prevention (CDC) 2009a]. These studies, along with more geographically targeted studies of pregnant women, show that pregnant women are also exposed to many chemicals (Bradman et al. 2003; Swan et al. 2005). Chemicals can cross the placenta and enter the fetus, and a number of chemicals measured in maternal urine and serum have also been found in amniotic fluid, cord blood, and meconium (Barr et al. 2007). In some cases, such as for mercury, fetal exposures may be higher than maternal exposure (Barr et al. 2007).

Multiple chemical exposures are of increasing concern. Studies show that exposure to multiple chemicals that act on the same adverse outcome can have a greater effect than exposure to an individual chemical. This has been recognized by the National Academy of Sciences (NAS), which recommends that future efforts accounting for risks from multiple chemical exposures combine effects from chemicals acting on the same adverse health outcome (National Research Council 2008a). Subsequently, assessment of exposure to multiple chemicals has been identified as an important future research area (Kortenkamp 2007).

Because few data are available on levels of individual or multiple chemicals in pregnant women, levels in reproductive-age women have often been used as an indicator of chemical levels in pregnant women (Blount et al. 2000). Some studies have directly compared pregnant women in their cohort and reproductive-age women from the National Health and Nutritional Examination Survey (NHANES), a nationally representative sample of the U.S. population. For example, phthalates measured in pregnant women from three U.S. locations were lower than those measured in reproductive-age women from NHANES (Swan et al. 2005). Numerous physiological changes occur during pregnancy, including weight gain and increases in blood and plasma volume, which can affect concentrations of chemicals (Chesley 1972; Pirani and Campbell 1973). Chemicals may also concentrate in the fetus, which could influence maternal concentrations (Takahashi and Oishi 2000). Further, behavioral changes occurring during pregnancy, such as diet modification (e.g., quantity and food type), may also influence chemical body burdens in pregnant women (Mirel et al. 2009). Understanding whether some of these factors can influence maternal concentrations of chemicals helps inform our ability to use measurements of chemicals in nonpregnant women as a surrogate for pregnant women.

We analyzed biomonitoring data for pregnant women from NHANES to characterize exposure to individual and multiple chemicals and their metabolites in pregnant women. Additionally, we evaluated the extent to which levels measured in nonpregnant women are representative of levels in pregnant women, and what factors may explain observed differences.

## Methods

### Study population

NHANES, conducted by the CDC, is a nationally representative survey and physical examination assessing the health and nutritional status of the civilian, noninstitutionalized U.S. population. The survey also includes measurement of chemicals and their metabolites in blood and urine (for more information, see CDC 2010). We use the term “chemical analyte” here to describe both chemicals and their metabolites. Because of the complex stratified survey design in NHANES, separate sample weights are assigned to each survey respondent; each participant represents approximately 50,000 other U.S. residents. Pregnant women were oversampled in the NHANES survey from 2001 to 2006 (CDC 2009b). [Protocols for oversampling pregnant women are described in Supplemental Material (doi:10.1289/ehp.1002727) and in detail elsewhere (Mirel et al. 2009).] We classified pregnancy status according to the results of the urine pregnancy test administered as part of NHANES protocols.

Most chemical analytes were measured in subsets of the total NHANES sample. Each subset included about one-third the total number of participants, so not all chemical analytes were measured in each participant. Further, not every group of chemical analytes was measured in each cycle. Therefore, we analyzed the 2003–2004 cycle, because it represents the cycle with the highest number of chemical analytes measured across the sample of pregnant women. We limited our study population to those 15–44 years of age to be consistent with the definition used by the National Center for Health Statistics for women of childbearing age (Chandra et al. 2005). Therefore, our study population includes 268 pregnant women and 1,489 nonpregnant women 15–44 years of age included in at least one subsample for chemical analyte analysis.

### Environmental chemical analyte analyses

Chemical analyte analyses were conducted at the National Center for Environmental Health laboratories (CDC, Atlanta, GA). Analytical procedures and summary statistics for the general population have been described in the Fourth National Report on Human Exposure to Environmental Chemicals and in the peer-reviewed literature (Calafat et al. 2008; Caldwell et al. 2009; CDC 2009a; Sjodin et al. 2008). We assessed 163 chemical analytes across 12 chemical classes (Table 1), measured in blood, urine, and serum.

### Data analysis

We conducted analyses in SUDAAN (version 10.0; Research Triangle Institute, Research Triangle Park, NC) and SAS (version 9.2; SAS Institute Inc., Cary, NC). SUDAAN calculates variance estimates after incorporating the nonrandom sampling design and the sample population weights, which account for oversampling of certain subgroups.

We examined summary statistics and distributional plots for each chemical analyte. We calculated the following descriptive statistics [for further details on analysis, see Supplemental Material (doi:10.1289/ehp.1002727)]: percentage of women with levels greater than the limit of detection (LOD), geometric mean (GM), geometric standard error (GSE), median and 95th percentile estimates, and the coefficient of variation (CV; defined as the GSE divided by the GM). The GM, GSE, and CV were calculated only for chemical analytes with > 60% detection frequency. The median and 95th percentile were calculated for all chemical analytes. Concentrations below the LOD were substituted by the CDC with 
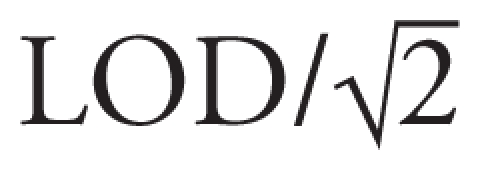
. We present statistical results for individual chemical analytes in the main text that are representative of each chemical class [for descriptive statistics and LODs for all 163 chemical analytes, see Supplemental Material, Table 1 (doi:10.1289/ehp.1002727)]. Representative chemical analytes were chosen based on public health relevance and expectation of relatively widespread exposure.

To assess extent of multiple exposures within a chemical class, we evaluated the number of individual PBDEs, perfluorinated compounds (PFCs), organochlorine pesticides, and phthalates detected in each pregnant woman. We chose these chemical classes to represent banned persistent chemicals (organochlorine pesticides), persistent chemicals (PBDEs and PFCs), and currently used nonpersistent chemicals (phthalates).

We then evaluated the extent of multiple chemical exposures across chemical classes in three different subsamples. These three subsamples were the primary subsamples of the pregnant women. Pregnant women in subsample A were assessed for metals, cotinine, and PFCs (17 chemical analytes in 76 women); in subsample B, for metals, cotinine, organochlorine pesticides, phthalates, PBDEs, and polycyclic aromatic hydrocarbons (PAHs) (52 chemical analytes in 54 women); and in subsample C, for metals, phenols, polychlorinated biphenyls (PCBs), organophosphate insecticide metabolites, perchlorate, and cotinine (71 chemical analytes in 59 women) [for subsample composition, see Supplemental Material, Table 2 (doi:10.1289/ehp.1002727)]. Volatile organic compounds (VOCs) were measured only in a subsample of pregnant women that partially overlapped with subsamples A, B, and C. Consequently, we did not include VOCs in analyses of multiple chemical exposures.

To compare chemical analyte concentrations between pregnant and nonpregnant women, we constructed multivariate regression models, which included our main effect (binary pregnancy status variable) along with covariates. We log-transformed chemical analytes before regression analysis to account for the nonnormal distributions. From these models, we calculated the least-squares geometric means (LSGMs), which provide GM estimates after adjustment for other covariates. For every chemical analyte in the main analysis, we used the same set of covariates. Covariates were included if they were significant predictors of more than one chemical analyte or if their inclusion in the model changed the β-coefficient for the main effect by > 20%. The following covariates were evaluated: age (continuous), race/ethnicity (Mexican American, non-Hispanic white, non-Hispanic black, or other), education (high school diploma or less vs. more than high school diploma), marital status (married/living with a partner, divorced/separated, or never married), parity (number of pregnancies resulting in live births, nulliparous vs. one or more child), current body mass index (BMI; continuous), smoking status (never, former, or current), serum albumin (continuous), length of food and drink fasting before blood collection (0–4.5 hr, 4.5–8.5 hr, or 8.5–24 hr), and urinary creatinine (continuous). All regression models were adjusted for the same covariates except for creatinine (included in models for urinary chemicals only). We excluded 12 nonpregnant women who reported fasting times > 24 hr. We defined statistical significance as *p* < 0.10 for all analyses because of relatively small number of pregnant women sampled for each chemical analyte and, consequently, small degrees of freedom.

As a sensitivity analysis, we performed multivariate regression in women < 35 years of age, because the age distribution differed between the two groups. For this analysis, we selected model covariates separately for each individual chemical analyte using the covariate selection method described above. Thus, the covariates in the sensitivity analysis may differ from that used in the main analysis. We conducted sensitivity analyses for lead (*n* = 215 pregnant; *n* = 885 nonpregnant), BPA (*n* = 63 pregnant; *n* = 275 nonpregnant), and *p*,*p*′-dichlorodiphenyldichloroethene (DDE) (*n* = 65 pregnant; *n* = 380 nonpregnant).

## Results

Although most pregnant and nonpregnant women were white, there was a higher percentage of Mexican-American pregnant women compared with nonpregnant women, reflecting higher birth rates among Hispanic women in the United States (Table 2) (Martin et al. 2007). Nonpregnant women were older, less likely to be married or with a partner, and more likely to smoke than were pregnant women (Table 2). In addition, pregnant women had lower levels of albumin and shorter fasting times before blood collection than did nonpregnant women.

Table 3 summarizes statistics for pregnant and nonpregnant women for select chemical analytes [for all 163 chemical analytes in pregnant women, see Supplemental Material, Table 1 (doi:10.1289/ehp.1002727)]. We found that 0–100% of pregnant women had a detectable level across the individual chemical analytes. Eight of 12 classes of chemicals included individual chemical analytes detected in 99–100% of pregnant women (PFCs, PBDEs, PCBs, organochlorine pesticides, phenols, phthalates, PAHs, and perchlorate). Four classes (VOCs, PFCs, PCBs, and organochlorine pesticides) included at least one individual chemical analyte not detected in any pregnant women [see Supplemental Material, Table 1 (doi:10.1289/ehp.1002727)]. In general, organophosphate metabolites, VOCs, and dioxins and furans were less frequently detected in pregnant women than were the other chemical classes except for dimethylthiophosphate (DMTP), toluene, *m*- and *p*-xylene, and methyl *tert*-butyl ether (MTBE).

Among pregnant women, DDE had the highest GM concentration (140.4 ng/g lipid) of the persistent, lipophilic compounds measured in serum (PCBs, PBDEs, and organochlorine pesticides), whereas concentrations of most of the other measured chemical analytes in these classes were an order of magnitude lower (PCBs, 4–8 ng/g lipid; PBDEs, 5–23 ng/g lipid). Perfluorooctane sulfonic acid (PFOS) had the highest GM among the persistent chemical analytes that do not accumulate in lipids (e.g., lead, cadmium, and PFCs). Of the nonpersistent chemical analytes measured in urine (organophosphate metabolites, phenols, phthalates, PAHs, and perchlorate), triclosan, benzophenone-3, and monoethyl phthalate (MEP) had the highest GMs (17.00, 25.49, and 226.53 μg/L, respectively).

Although the GM for cotinine was < 1 μg/L, the range of concentrations spanned three orders of magnitude (CV = 0.31). Variability in other chemical analyte levels measured in pregnant women was generally low (CV < 0.25), except for some phenols (CV = 0.25–0.51), phthalates (CV = 0.22–0.35), MTBE (CV = 0.40), triclosan (CV = 0.51), and PBDE-153 (CV = 0.31).

**Figure 1:**
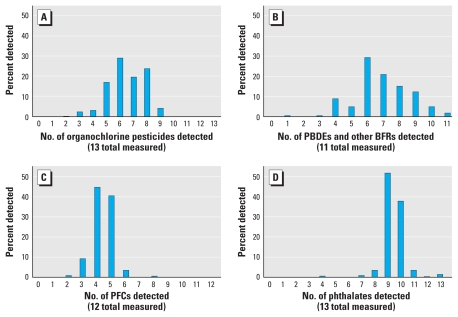
Distribution of the number of chemicals detected in U.S. pregnant women for four chemical classes: organochlorine pesticides (*A; n* = 71), PBDEs (*B; n* = 75), PFCs (*C; n* = 76), and phthalates (*D; n* = 91).

Figure 1 shows the numbers of individual PFC, PBDE, organochlorine pesticide, and phthalate chemical analytes detected in individual pregnant women. At least two organochlorine pesticides, one PBDE, two PFCs, and four phthalates were measured in each pregnant woman. The median number of chemicals detected for organochlorine pesticides, PBDEs, PFCs, and phthalates were 6, 6, 4, and 9, respectively. For PBDEs and phthalates, 7% and 2%, respectively, had detectable levels of ≥ 90% of the chemical analytes in the class.

**Figure 2:**
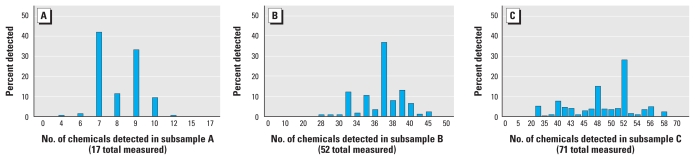
Distribution of the number of chemicals detected in U.S. pregnant women across multiple chemical classes. (*A*) Subsample A (metals, cotinine, and PFCs). (*B*) Subsample B (metals, cotinine, organochlorine pesticides, phthalates, PBDEs, and PAHs). (*C*) Subsample C (metals, phenols, PCBs, organophosphate insecticide metabolites, perchlorate, and cotinine).

**Figure 3:**
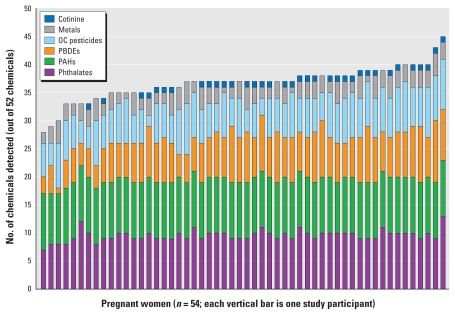
Number of chemicals detected by chemical class in U.S. pregnant women, NHANES subsample B [metals, cotinine, organochlorine (OC) pesticides, phthalates, brominated flame retardants (PBDEs), and PAHs], 2003–2004 (*n* = 54). Each vertical bar represents one study participant. Other subsamples showed similar results.

The median number of chemical analytes detected among women in subsamples A, B, and C were 8 (range, 4–12), 37 (range, 28–45), and 50 (range, 35–60), respectively (Figure 2). We found generally that the overall number of chemicals detected was not dominated by detects within a particular chemical class (Figure 3). For example, several participants in subsample B at the median detected level (37 chemicals) had 10 phthalates, 10 PAHs, 7 PBDEs, 6 organochlorine pesticides, 3 metals, and cotinine detected.

GM and median levels for most chemicals were similar to or lower than those in pregnant than in nonpregnant women, except for PBDEs, DMTP, triclosan, and perchlorate (Table 3). About half the LSGM estimates for pregnant women (Table 4) increased after adjusting for covariates (Tables 3 and 4). For a few chemicals, the LSGM estimates for pregnant women decreased after adjustment, such as PBDEs, some phthalates, perchlorate, and BPA. In general, adjusted LSGMs were comparable between pregnant and nonpregnant women (Table 4). Nonpregnant women had significantly higher levels of cadmium, lead, PFOS, BPA, and cotinine, but pregnant women had significantly higher levels of DDE, DMTP, MTBE, and perchlorate (Table 4). The most pronounced differences between pregnant and nonpregnant women were for MTBE and DMTP (levels in pregnant women were about two times those of nonpregnant women) and cotinine (levels in pregnant women were about half those of nonpregnant women).

Serum albumin influenced the comparison between pregnant and nonpregnant women for 28 of the 32 compounds evaluated in regression analyses (the β-coefficient changed by > 20%); however, direction of the effect varied by type of compound. In general, for chemical analytes measured in blood, effect estimates for albumin were positive, and their inclusion increased the LSGMs for pregnant women; in contrast, for nonpersistent urinary chemical analytes, the albumin effect estimates were more often negative, and their inclusion decreased the LSGMs for pregnant women (data not shown). Smoking influenced comparison of LSGMs between pregnant and nonpregnant women for 75% of chemicals. Maternal age and BMI changed the LSGMs for persistent organic pollutants such as PCBs, and creatinine influenced LSGMs for most chemical analytes measured in urine. Other variables, such as race/ethnicity and education, were often significant predictors of chemical analyte concentrations but generally did not change LSGM comparisons in Table 4.

Compared with estimates based on women of all ages, LSGMs for lead and DDE for both pregnant and nonpregnant women were reduced when we restricted analyses to younger women (< 35 years of age). However, relative differences in adjusted estimates between pregnant and nonpregnant women were not substantially affected. LSGMs for BPA increased for both groups in the restricted analysis, and the differences in LSGM estimates between pregnant and nonpregnant women were no longer statistically significant [LSGM = 2.16 (pregnant) vs. 3.03 μg/L (nonpregnant), *p* = 0.24].

## Discussion

We found widespread exposure to pregnant women in the United States to multiple chemical analytes, including both banned and contemporary contaminants. Although we did not make any direct connection to potential adverse health consequences, levels of many of these chemical analytes were similar to those measured in epidemiologic studies finding an association between prenatal chemicals exposure and adverse reproductive and developmental outcomes. These include phthalates and increased risk of adverse male reproductive outcomes (Swan et al. 2005), mercury and developmental neurological outcomes (Lederman et al. 2008), PBDEs and neurodevelopmental outcomes (Herbstman et al. 2010), and PCBs and maternal thyroid hormone disruption during pregnancy (Chevrier et al. 2008).

Additionally, pregnant women were exposed to multiple chemical analytes at one time, many of which can affect the same adverse outcomes. Examples include maternal thyroid hormone disruption [e.g., perchlorate, PCBs, PBDEs, and triclosan (Crofton 2008)], male reproductive development (multiple phthalates), and the developing brain (mercury, lead, PCBs) (National Research Council 2008a). The NAS has recommended risk assessment of multiple chemicals expand to account for chemicals acting on a common adverse outcome (National Research Council 2008a). Although the NAS focused on grouping chemicals contributing to disturbances of androgen action, they also proposed this approach for chemicals affecting brain development (National Research Council 2008a).

Levels of chemicals measured during pregnancy can be influenced by physiological (e.g., changes in BMI, plasma volume expansion, and bone mobilization) and behavioral factors. For example, previous research has found an inverse relationship between weight gain during pregnancy and levels of persistent organic pollutants in pregnant women (Bradman et al. 2006). We found that plasma volume expansion, using the level of albumin as a surrogate, may also influence chemical levels measured in pregnant women. Plasma volume begins to expand in pregnant women at around 8 weeks of gestation and increases progressively until 30–34 weeks gestation, when it plateaus. This expansion may dilute environmental chemical concentrations in blood (Faupel-Badger et al. 2007). Accurately measuring plasma volume expansion is expensive and ideally requires multiple measurements throughout pregnancy (Faupel-Badger et al. 2007). However, albumin measurements may provide a reasonable surrogate because previous studies suggest that blood volume expansion dilutes circulating levels of albumin during pregnancy (Honger 1968). We found that, in general, adjusting for albumin increased GM estimates of persistent compounds, such as DDE, in pregnant women, suggesting that the concentration is diluted by increased plasma volume. However, adjustment for albumin generally decreased estimates for nonpersistent compounds, such as BPA, in pregnant women, suggesting that lower albumin may be associated with an increased clearance of environmental contaminants. Albumin may affect metabolism and transport of chemicals by mechanisms other than plasma volume expansion. For example, previous research has shown that PFCs actually bind to albumin in the blood (Jones et al. 2003). BPA also binds to plasma proteins, such as albumin, in humans (Teeguarden et al. 2005), so reduced albumin during pregnancy may influence the amount of BPA that undergoes phase II conjugation and subsequent elimination through urine. The role of albumin, and other transport proteins, in the transport and metabolism of environmental chemicals, particularly during pregnancy, is an important topic and requires further research.

We found that, generally, the levels in pregnant women were similar to or lower than levels measured in nonpregnant women. Adjusting for physiological factors that may influence levels of chemicals in pregnant women tended to increase the levels in pregnant women compared with nonpregnant women. This suggests that generally levels of chemicals in nonpregnant reproductive-age women are reasonably representative of levels found in pregnant women. However, for several chemicals, levels in pregnant women remain lower than those in nonpregnant women. Behavioral factors may explain this difference (e.g., cotinine and smoking), or other physiological factors may be important [e.g., chemical levels concentrating in the fetus such as for BPA (Takahashi and Oishi 2000)].

The NHANES study design, where groups of chemicals were analyzed in approximate one-third–sized subsamples, meant that we could not evaluate more than 71 chemical analytes in any individual pregnant women, or about 44% of chemical analytes measured during 2003–2004. This also limited our ability to assess exposures to multiple chemical analytes that may be acting on the same adverse outcome (e.g., PBDEs and PCBs, which can affect neurodevelopment, were not measured in the same women). Given that several chemical analytes within each of the classes were detected almost ubiquitously, pregnant women have more detectable chemical analytes than we could assess in any individual participant in this analysis.

Other methodological changes between cycles make it challenging to compare data across NHANES cycles. For example, the number and types of chemicals sampled changes by cycle. Another challenge is that LODs vary among the cycles. Mostly they decreased, such as with PCBs, which can increase the number of chemicals detected. However, a few LODs increased; for example, certain urinary phthalate esters, such as mono-2-ethylhexyl phthalate (MEHP) and MEP, increased between 2003–2004 and 2005–2006.

Chemical analyte concentrations in NHANES participants should be representative of typical U.S. concentrations. Thus, highly exposed subpopulations may be underrepresented. For example, women living in the agricultural Salinas Valley of California had higher measurable levels of several pesticides than did NHANES pregnant women (Castorina et al. 2010). Other subpopulations may have nonrepresentative exposure patterns, such as high fish consumption or higher use of certain personal care products.

Our analysis indicates high variability in exposures for some chemical analytes, shown by the relatively high CV for phenols, phthalates, cotinine, and MTBE. For some of these analytes, with almost an order of magnitude difference between the median and the 95th percentile, variation may reflect geographic variability in exposure sources. For example, MTBE was used in reformulated gasoline starting in 1995. Reformulated gasoline was required for use year-round in cities with significant smog problems (Energy Information Administration 2008), so it was not used in every U.S. location. Thus, the geographic variation in MTBE use may play a role in the wide exposure variability (Energy Information Administration 2008). PBDE-153 is another example of how geographic use variation can influence exposures levels. The 95th percentile of PBDE-153 levels is 15 times greater than the median, and previous research has found PBDE concentrations to be around two times higher in Californians than in others in the United States, likely because of California’s unique flammability standard (Zota et al. 2008). Variation in exposure to chemical analytes used in consumer and personal care products (e.g., triclosan, where the 95th percentile is 35 times greater than the median) could be driven by unique product uses (Allmyr et al. 2009). Although biomonitoring studies can demonstrate variation in exposures within populations, they generally are limited in their ability to identify sources of exposures. Consequently, additional exposure assessment research is needed to identify the dominant sources of exposure among pregnant women and the general population.

Our analysis of the NHANES pregnancy data shows ubiquitous exposure to multiple chemicals during a sensitive period of fetal development. The NAS recommends accounting for both multiple exposures and exposures that occur during vulnerable developmental periods in improved approaches for assessing chemical risks across the population, which includes shifting to a risk assessment approach that presumes no threshold of effect among the population unless shown otherwise (National Research Council 2008b). Data, such as from NHANES, should be used to enhance our understanding of risks among the U.S. population and to inform further policy and research activities.

## Figures and Tables

**Table 1:** Chemical classes measured in biological tissue of pregnant women, NHANES 2003–2004.

	No. of chemical analytes measured			
Chemical class	Blood	Serum	Urine	Total
Cotinine		1		1

Environmental phenols			4	4

Metals	4			4

Organochlorine pesticides		13		13

Organophosphate insecticides			6	6

Perchlorate			1	1

Phthalates			13	13

PBDEs and other brominated flame retardants		11		11

PCBs and dioxin-like chemicals		55		55

PAHs			10	10

PFCs		12		12

VOCs	33			33

See Supplemental Material, Table 1 (doi:10.1289/ehp.1002727), for individual chemical analytes included in each chemical class.

**Table 2:** Characteristics of reproductive-age women by pregnancy status, NHANES 2003–2004.

Demographic characteristic	Pregnant women (*n* = 268)	Nonpregnant women (*n* = 1,489)
Age [years (mean ± SE)]**	27 ± 0.8	30 ± 0.37

Age [years (%)]**		
15–17	4	10
18–24	30	23
25–29	31	13
30–34	25	17
35–44	11	37

Race/ethnicity (%)**		
Non-Hispanic white	56	67
Non-Hispanic black	18	14
Mexican American	17	10
Other Hispanic	2	5
Other	6	5

Education (%)		
< High school diploma	26	24
High school diploma	15	22
> High school diploma	59	54

Marital status (%)**		
Married or living with partner	79	50
Divorced, separated, or widowed	2	12
Never married	19	38

Parity (%)**		
0	45	44
1	34	14
≥ 2	21	42

Smoking status (%)**		
Never	59	60
Former	31	11
Current	9	30

Trimester		
First	31	
Second	32	
Third	37	

Biochemical measurements		
Serum albumin [g/dL (mean ± SE)]**	3.46 ± 0.04	4.23 ± 0.01
Urinary creatinine [mg/dL (mean ± SE)]	127.81 ± 6.00	130.86 ± 3.27

Sampling characteristics		
Duration of food and drink fasting before blood collection [hr (mean ± SE)]**	8.40 ± 0.73	10.67 ± 0.10

Data were missing in pregnant women for parity (*n* = 18), education (*n* = 3), smoking (*n* = 6), trimester (*n* = 41), and length of fasting (*n* = 2) and in nonpregnant women for parity (*n* = 160), education (*n* = 46), smoking (*n* = 151), and length of fasting (*n* = 25).

** *p* < 0.01.

**Table 3:** Descriptive statistics for chemical analytes in pregnant and nonpregnant women, NHANES 2003–2004.^a^

Chemical analyte	*n*	Reproductive status	LOD^a^	Percent > LOD	GM (GSE)	50th percentile	95th percentile	CV
Metals [blood (μg/L)]								
Cadmium**	253	Pregnant	0.14	66	0.22 (0.01)	0.2	0.8	0.07
	1,396	Nonpregnant		79	0.33 (0.01)	0.3	1.6	0.03

Lead (μg/dL)**	253	Pregnant	0.28	94	0.68 (0.04)	0.6	1.8	0.06
	1,396	Nonpregnant		99	0.96 (0.04)	0.9	2.4	0.04

Mercury (total)*	253	Pregnant	0.20	89	0.67 (0.07)	0.7	3.4	0.10
	1,396	Nonpregnant		92	0.80 (0.05)	0.8	4.4	0.06

VOCs [blood (μg/L)]								
Benzene	89	Pregnant	0.024	38	—b	< LOD	0.2	—b
	389	Nonpregnant		53	—b	< LOD	0.3	—b

1,4-Dichlorobenzene	89	Pregnant	0.12	40	—b	< LOD	20.0	—b
	373	Nonpregnant		47	—b	< LOD	4.1	—b

MTBE (methyl *tert*-butyl ether)	85	Pregnant	0.002	86	0.01 (0.01)	0.02	0.1	0.40
	373	Nonpregnant		78	0.01 (0.002)	0.01	0.1	0.20

Toluene**	90	Pregnant	0.025	94	0.07 (0.01)	0.1	0.2	0.07
	387	Nonpregnant		95	0.10 (0.01)	0.1	0.5	0.10

Cotinine [serum (μg/L)]**	249	Pregnant	0.015	66	0.07 (0.02)	0.03	68.8	0.31
	1,369	Nonpregnant		83	0.54 (0.13)	0.1	318.0	0.24

PFCs [serum (μg/L)]								
Perfluorooctanoic acid*	76	Pregnant	0.1	99	2.39 (0.24)	2.6	5.6	0.10
	400	Nonpregnant		99	3.19 (0.16)	3.2	8.4	0.05

PFOS (perfluorooctanyl sulfonate)**	76	Pregnant	0.4	99	12.29 (1.02)	12.0	21.8	0.08
	400	Nonpregnant		100	16.26 (0.84)	15.5	44.0	0.05

PBDEs [serum (ng/g lipid)]								
PBDE-47	75	Pregnant	4.2	99	23.90 (2.21)	23.7	100.0	0.09
	441	Nonpregnant		98	21.15 (2.03)	21.2	114.0	0.10

PBDE-99	75	Pregnant	5.0	87	5.51 (0.81)	5.1	21.8	0.15
	434	Nonpregnant		68	5.04 (0.42)	4.4	31.5	0.08

PBDE-100*	75	Pregnant	1.4	99	6.06 (0.91)	6.6	23.2	0.15
	443	Nonpregnant		96	4.00 (0.43)	3.8	25.2	0.11

PBDE-153	75	Pregnant	2.2	100	9.90 (3.04)	7.8	127.0	0.31
	442	Nonpregnant		93	5.18 (0.53)	4.5	43.9	0.10

PCBs [serum (ng/g lipid)]								
PCB-118	75	Pregnant	0.6	100	4.31 (0.95)	3.6	14.3	0.22
	415	Nonpregnant		100	4.46 (0.28)	4.3	16.9	0.06

PCB-138 and -158	75	Pregnant	0.4	100	7.70 (1.24)	7.3	20.2	0.16
	416	Nonpregnant		100	8.95 (0.55)	8.3	37.0	0.06

PCB-153	75	Pregnant	1.1	100	8.74 (1.29)	8.8	22.5	0.15
	415	Nonpregnant		100	11.07 (0.64)	10.2	44.0	0.06

PCB-180*	75	Pregnant	0.4	96	4.61 (0.99)	6.8	13.2	0.21
	416	Nonpregnant		99	7.42 (0.44)	7.5	33.3	0.06

Organochlorine pesticides [serum (ng/g lipid)]								
DDT (dichlorodiphenyltrichloroethane)	71	Pregnant	7.8	62	—c	—c	37.4	0.16
	426	Nonpregnant		63	—c	—c	13.3	0.06

DDE (dichlorodiphenyldichloroethylene)	71	Pregnant	7.8	100	140.39 (29.72)	99.9	850.0	0.21
	424	Nonpregnant		99	151.04 (16.03)	141.0	815.0	0.11

Hexachlorobenzene*	70	Pregnant	7.8	100	11.27 (1.08)	10.4	25.7	0.10
	428	Nonpregnant		99	14.34 (0.39)	14.3	25.7	0.03

Organophosphate insecticide metabolites [urine (μg/L)]								
Dimethylphosphate	89	Pregnant	0.5	44	—b	< LOD	13.7	—b
	483	Nonpregnant		48	—b	< LOD	14.3	—b

Diethylphosphate	89	Pregnant	0.1	33	—b	< LOD	10.8	—b
	474	Nonpregnant		49	—b	< LOD	14.8	—b

DMTP*	89	Pregnant	0.5	83	2.43 (0.43)	2.7	16.0	0.18
	483	Nonpregnant		73	1.81 (0.17)	1.7	28.3	0.09

Diethylthiophosphate	87	Pregnant	0.2	57	—b	0.2	2.2	—b
	478	Nonpregnant		46	—b	< LOD	2.6	—b

Dimethyldithiophosphate	86	Pregnant	0.1	56	—b	0.2	3.2	—b
	475	Nonpregnant		34	—b	< LOD	4.0	—b

Environmental phenols [urine (μg/L)]								
BPA	86	Pregnant	0.4	96	2.53 (0.63)	2.7	15.0	0.25
	489	Nonpregnant		96	2.89 (0.29)	3.0	17.6	0.10

Triclosan	86	Pregnant	2.3	87	17.00 (8.74)	8.2	283.0	0.51
	489	Nonpregnant		81	14.65 (0.97)	11.1	411.0	0.07

Benzophenone-3	86	Pregnant	0.3	100	25.49 (6.52)	16.9	353.0	0.26
	489	Nonpregnant		98	37.14 (6.44)	31.4	1530.0	0.17

Phthalates [urine (μg/L)]								
Monobenzyl phthalate	91	Pregnant	0.1	100	15.12 (3.79)	17.8	86.8	0.25
	497	Nonpregnant		100	14.77 (0.79)	15.5	99.9	0.05

Monoisobutyl phthalate	91	Pregnant	0.3	99	3.47 (0.84)	4.4	19.5	0.24
	497	Nonpregnant		98	4.21 (0.27)	4.5	21.1	0.06

Mono-*n*-butyl phthalate	91	Pregnant	0.4	99	18.83 (4.11)	17.1	143.8	0.22
	497	Nonpregnant		99	24.64 (1.16)	25.7	132.2	0.05

MEP	91	Pregnant	0.4	100	226.53 (79.03)	265.7	2263.0	0.35
	497	Nonpregnant		100	246.06 (29.56)	234.5	2992.6	0.12

PAHs [urine (μg/L)]								
9-Hydroxyfluorene	85	Pregnant	0.005	100	0.21 (0.04)	0.2	0.8	0.19
	478	Nonpregnant		100	0.30 (0.03)	0.2	1.1	0.11

2-Naphthol	91	Pregnant	0.031	100	2.49 (0.59)	2.4	14.7	0.24
	492	Nonpregnant		100	3.68 (0.31)	3.3	28.7	0.08

2-Hydroxyphenanthrene	87	Pregnant	0.005	100	0.06 (0.01)	0.05	0.2	0.17
	479	Nonpregnant		99	0.06 (0.004)	0.06	0.3	0.07

1-Hydroxypyrene	86	Pregnant	0.005	100	0.08 (0.02)	0.08	0.5	0.25
	481	Nonpregnant		99	0.09 (0.007)	0.09	0.6	0.07

Perchlorate [urine (μg/L)]*	89	Pregnant	0.05	100	4.17 (0.84)	4.3	34.0	0.07
	492	Nonpregnant		100	2.68 (0.21)	2.8	11.0	0.08

a For most chemicals, the LOD is constant across samples. However, for persistent organic pollutants (PBDEs, PCBs, and organochlorine pesticides), each individual sample has its own LOD because the available sample volume differed by sample, and a higher sample volume results in a lower LOD. For chemicals with sample-specific LODs, the maximum LOD is reported. In general, the average LOD is approximately 40–50% of the maximum LOD (CDC 2009).

b GM,GSE, or CV could not be calculated because detection frequency is< 60%.

c GM or percentile estimate is not reported because it is less than the maximum LOD.

* *p* < 0.10;

** *p* < 0.01; calculated using univariate regression analysis.

**Table 4:** Comparison of chemical analyte concentrations between pregnant and nonpregnant women after adjustment for covariates,^a^ calculated from multivariate regression models.

		Pregnant women		Nonpregnant women	
Chemical analyte	β-Coefficient (90% CI)^b^	LSGM	90% CI	LSGM	90% CI
Metals [blood (μg/L)]		*n* = 225		*n* = 1,091	

Cadmium	−0.20 (−0.36 to −0.04)*	0.27	0.23–0.31	0.33	0.31–0.35
Lead (μg/dL)	−0.16 (−0.27 to −0.06)*	0.80	0.72–0.89	0.94	0.89–0.99
Mercury (total)	−0.11 (−0.33 to 0.10)	0.71	0.57–0.89	0.79	0.72–0.88

VOCs [blood (μg/L)]		*n* = 82		*n* = 334	

MTBE	0.97 (0.03 to 1.90)*	0.02	0.01–0.06	0.008	0.005–0.01
Toluene	0.15 (−0.14 to 0.43)	0.11	0.08–0.14	0.09	0.08–0.10

Cotinine [serum (μg/L)]		*n* = 225		*n* = 1,091	

	−0.94 (−1.39 to −0.48)**	0.19	0.13–0.28	0.49	0.42–0.58

PFCs [serum (μg/L)]		*n* = 70		*n* = 313	

Perfluorooctanoic acid	−0.18 (−0.37 to 0.02)	2.69	2.18–3.32	3.22	2.95–3.52
PFOS	−0.23 (−0.35 to −0.12)**	12.81	11.94–13.74	16.28	15.18–17.46

PBDEs [serum (ng/g lipid)]		*n* = 68		*n* = 366	

PBDE-47	0.02 (−0.32 to 0.35)	21.76	16.73–28.30	21.33	18.21–24.97
PBDE-99	−0.11 (−0.47 to 0.26)	4.62^c^	3.37–6.33	5.10	4.44–5.87
PBDE-100	0.24 (−0.22 to 0.70)	5.21	3.60–7.52	4.10	3.38–4.97
PBDE-153	0.51 (−0.10 to 1.12)	8.85	5.05–15.50	5.31	4.46–6.33

PCBs [serum (ng/g lipid)]		*n* = 66		*n* = 334	

PCB-118	−0.02 (−0.31 to 0.28)	4.39	3.20–6.02	4.44	3.99–4.93
PCB-138 and -158	−0.07 (−0.33 to 0.19)	8.25	6.57–10.36	8.85	7.96–9.83
PCB-153	−0.11 (−0.39 to 0.17)	9.87	7.73–12.62	11.02	9.92–12.25
PCB-180	−0.27 (−0.65 to 0.11)	5.64	3.97–8.01	7.39	6.77–8.07

Organochlorine pesticides [serum (ng/g lipid)]		*n* = 64		*n* = 354	

DDT	−0.10 (−0.32 to 0.13)	3.49^c^	2.78–4.38	3.86^c^	3.60–4.14
DDE	0.33 (0.12 to 0.53)*	198.34	160.72–244.78	142.59	126.13–161.21
Hexachlorobenzene	−0.02 (−0.14 to 0.10)	13.74	12.36–15.26	14.01	13.53–14.51

Organophosphate insecticide metabolites [urine (μg/L)]		*n* = 74		*n* = 370	

DMTP	0.85 (0.34 to 1.35)*	4.39	2.74–7.05	1.88	1.60–2.20

Environmental phenols [urine (μg/L)]		*n* = 72		*n* = 371	

BPA	−0.55 (−0.97 to −0.13)*	1.63	1.13–2.36	2.83	2.42–3.31
Triclosan	0.47 (−0.60 to 1.54)	23.81	8.17–69.36	15.03	13.06–17.29
Benzophenone-3	−0.07 (−1.26 to 1.12)	38.09	14.02–103.46	40.85	29.28–57.00

Phthalates [urine (μg/L)]		*n* = 75		*n* = 377	

Monobenzyl phthalate	−0.02 (−0.53 to 0.50)	14.73	8.86–24.49	15.03	13.77–16.41
Monoisobutyl phthalate	−0.37 (−0.76 to 0.03)	2.83	1.89–4.23	4.06	3.65–4.50
Mono-*n*-butyl phthalate	−0.26 (−0.62 to 0.11)	18.36	12.93–26.07	23.81	21.81–25.99
MEP	−0.13 (−0.93 to 0.66)	221.41	98.85–495.90	254.68	206.36–314.30

PAHs [urine (μg/L)]		*n* = 74		*n* = 372	

9-Hydroxyfluorene	−0.15 (−0.50 to 0.19)	0.20	0.14–0.28	0.23	0.21–0.26
2-Naphthol	−0.15 (−0.57 to 0.27)	3.00	1.97–4.58	3.49	3.20–3.81
2-Hydroxyphenanthrene	−0.12 (−0.27 to 0.02)	0.05	0.04–0.06	0.06	0.05–0.06
1-Hydroxypyrene	−0.14 (−0.46 to 0.19)	0.08	0.06–0.10	0.09	0.08–0.09

Perchlorate [urine (μg/L)]		*n* = 74		*n* = 374	

	0.25 (0.05 to 0.45)*	3.35	2.67–4.21	2.61	2.31–2.95

CI, confidence interval. Sample sizes for chemical classes are approximate because sample sizes vary slightly by chemical.

a Models adjusted for age, race/ethnicity, education, smoking, parity, BMI, albumin, duration of fasting before specimen collection, and creatinine (only urinary chemical analytes adjusted for creatinine).

b Reference group is nonpregnant women. Chemical analyte concentrations are log-transformed.

c LSGM (least-squares geometric mean) estimates are < LOD (see Table 3).

* *p* < 0.10;

** *p* < 0.01.
